# Peanut allergy in France: preliminary results of the MIRABEL pilot study

**DOI:** 10.1186/2045-7022-3-S3-P131

**Published:** 2013-07-25

**Authors:** VM Nguyen-Grosjean

**Affiliations:** 1 Allergy Vigilance Network, Vandoeuvre les Nancy, France; 2 Allergy Department, Hospital E Dürkheim, Epinal, France

## Background

Peanut allergy may affect 0.65% of the French population [1]. Analysis of risk, benefits and cost is the MIRABEL ongoing coordinated study, aiming to make the connection between medical data and the food consumptions of peanut allergic patients. The aim of this pilot study is the evaluation of feasibility.

## Methods

Concerned patients were volunteers, recruited during medical consultation to allergists.

An anonymous medical questionnaire was filled after informed consent. Symptoms, type of foods, reactive quantities, prick tests, anti-Ara h2 IgEs [2], oral challenges (OC) were required. A questionnaire for consumptions was filled by the patients at home.

## Results

Out of 61 patients, 66% were male, aged from 1 to 11 at the time of their first allergic reaction (median: 3 years). Peanut allergy was documented in 52 patients. Sensitization was observed in 9 cases. 95% of patients had atopy: atopic dermatitis (62.3%), asthma (55.7%), allergic rhinitis (47.5%). Associated food sensitizations or allergies were: tree nuts (47.7%), legumes (36.4%), egg (25%),other foods (34.1%). Symptoms were urticaria and/or angioedema (54.1%), digestive symptoms (19.7%), laryngeal angioedema (9.8%), asthma (6.5%), conjunctivitis (5.8%), atopic dermatitis (3.8%) serious systemic reaction (8.3), anaphylactic shock (1.6%). Exposition routes were ingestion (84.6%), skin contact (28.8%), inhalation (5.8%). Foods involved were roasted peanuts (59.6%), Curly® (34.6%), aperitive biscuits (17.3%) and others. The reactive amount was quantified in 56% of cases and was infinitesimal in 14.7% of cases. The median of the reactive amount was 102 mg (range:34-1062) of protein equivalent. A strict avoidance needing to read the labels was prescribed in 32.2% of cases, based on a low reactive threshold: median 24 mg (range:4.7-109 mg). Authorized traces were correlated to a higher reactive threshold: median 638 (range: 88-2217) (p=0.005). 50% of questionnaires for consumptions were sent back.

## Conclusion

The high predictive value of anti Ara h2 > 0.23 kU/L is confirmed and shows that the choice of a strict or non-strict avoidance diet is based on the reactive dose of oral challenge. The feasibility of the study is confirmed.

## Disclosure of interest

None declared.
